# Bactericidal antibodies against hypervirulent *Neisseria meningitidis* C field strains following MenC-CRM or MenACWY-CRM priming and MenACWY-CRM booster in children

**DOI:** 10.1080/21645515.2020.1833578

**Published:** 2020-12-16

**Authors:** Marzia Monica Giuliani, Alessia Biolchi, Pavitra Keshavan, Maria Moriondo, Sara Tomei, Laura Santini, Elena Mori, Alessandro Brozzi, Margherita Bodini, Francesco Nieddu, Silvia Ricci, Thembile Mzolo, Marco Costantini, Chiara Azzari, Michele Pellegrini

**Affiliations:** a Preclinical Evidence Generation, GSK, Siena, Italy; b Data Science and Digital Innovation, GSK, Siena, Italy; c Clinical Research and Development, GSK, Siena, Italy; d Biostat and Statistical Programming, GSK, Siena, Italy; e Department of Health Sciences, University of Florence and Meyer Children’s University Hospital, Florence, Italy; f Biostat and Statistical Programming, GSK, Amsterdam, The Netherlands

**Keywords:** Hypervirulent MenC strains, cc11/cc334 clonal complexes, outbreak, MenACWY-CRM conjugate vaccine, MenC-CRM conjugate vaccine

## Abstract

An increase in invasive meningococcal disease (IMD) incidence was observed in Tuscany in 2015/2016, mainly due to hypervirulent clonal complex (cc) 11 strains. In a *post-hoc* analysis, we assessed bactericidal activity of antibodies in sera from children primed with MenACWY-CRM or MenC-CRM conjugate vaccines and receiving a MenACWY-CRM booster dose against 5 meningococcal C (MenC) strains isolated from IMD cases. Sera collected from 90 infants/toddlers who participated in a phase III, open-label study (NCT00667602) and its extension (NCT01345721) were tested by serum bactericidal activity assay with human complement (hSBA). Children were primed with either MenACWY-CRM at 6–8 and 12 months of age (group 2_MenACWY; N = 30), MenACWY-CRM (group 1_MenACWY; N = 30), or MenC-CRM at 12 months of age (group 1_MenC; N = 30); all received MenACWY-CRM booster dose at 22–45 months of age. Four tested strains (FI001–FI004) were C:P1.5–1,10-8:F3-6:ST-11 (cc11) and 1 (FI005) was C:P1.7–4,14-6:F3-9:ST-1031 (cc334). Overall, immune responses tended to be higher against Fl002–FI004 than Fl001 and Fl005. Geometric mean titers were high in group 2_MenACWY (range: 94.8 [FI005]–588.1 [FI004]) and very high post-boosting with MenACWY-CRM in all groups (176.9 [FI005]–3911.0 [FI004]). Seroresponse rates tended to be higher in group 1_MenC (33.3% [FI005]–93.3% [FI004]) than in group 1_MenACWY (16.7% [FI005]–73.3% [FI004]). Irrespective of strains tested or the identity/number of priming doses, ≥96.7% of children had hSBA titers ≥1:8 post-MenACWY-CRM booster dose. MenACWY-CRM and MenC-CRM elicited bactericidal antibodies and immunological memory against hypervirulent cc11 and cc334 MenC strains responsible for IMD outbreaks.

## Introduction

An increase of *Neisseria meningitidis* serogroup C (MenC) invasive meningococcal disease (IMD) was reported during January 2015–February 2016 in Tuscany, a region located in the center of Italy.^1^ Within this period of slightly more than 1 year, 43 confirmed MenC IMD cases, of which 10 fatal, were reported, accounting for approximately 38% of all confirmed MenC IMD cases recorded since 2000 in the region (overall, 111 MenC IMD cases from January 2000 to February 2016). The vast majority (87.5%) of MenC strains isolated during this outbreak belonged to the clonal complex (cc) 11, with cc334 isolated in fewer individuals.^1^

**Figure 1.**
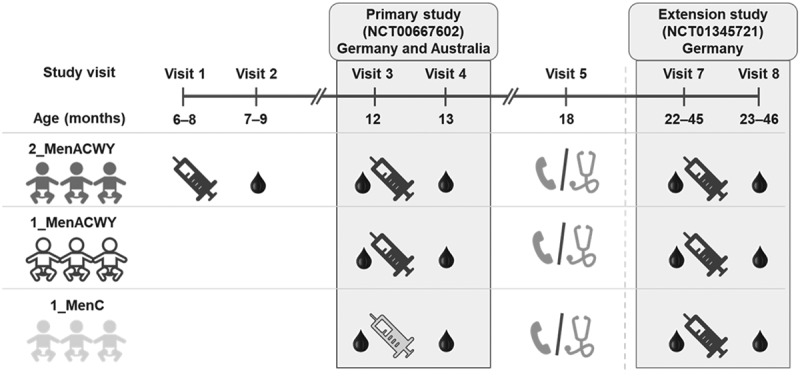
Study design for parent and extension studies

Group 2_MenACWY, MenACWY-CRM vaccination at 6–8 and 12 months of age; Group 1_MenACWY, MenACWY-CRM vaccination at 12 months of age; Group 1_MenC, MenC-CRM vaccination at 12 months of age. All children received a booster dose of MenACWY-CRM at 22–45 months of age.Note: Visits enclosed in gray boxes correspond to timepoints considered in the analysis. Syringes indicate vaccine administration (black for MenACWY-CRM and gray for MenC-CRM).Visit 3 was pre-second dose for group 2_MenACWY and pre-first dose for groups 1_MenACWY and 1_MenC; Visit 4 was post-primary vaccination, Visit 7, pre-booster vaccination, Visit 8, post-booster vaccination. Visit 6 (blood sample collection to evaluate persistence of immune response at 6–18 months post-primary vaccination) applied only to children enrolled at the Australian sites.

This *post-hoc* analysis aimed to evaluate the magnitude of immune responses against hypervirulent cc11 and cc334 strains in sera collected from infants/toddlers after priming with a monovalent MenC conjugate vaccine (MenC-CRM; *Menjugate*, GSK) or a quadrivalent MenA, MenC, MenW, MenY conjugate vaccine (MenACWY-CRM; *Menveo*, GSK) and a booster dose of MenACWY-CRM.

## Methods

### Post-hoc analysis methodology

Two completed studies, part of the clinical development program of MenACWY-CRM, were selected for this analysis, based on the population evaluated (infants/toddlers, at high risk of IMD) and the vaccine schedules (priming with different vaccines and doses, and assessment of booster responses). The first one was a phase III, open label, randomized, multicenter study (NCT00667602) performed in Germany and Australia in 2011. This study was conducted to evaluate immune responses and safety after priming of infants/toddlers with 1 or 2 doses of MenACWY-CRM, as compared to priming with a single dose of MenC-CRM. The second selected study was its extension, a phase IIIb trial (NCT01345721) performed in Germany to assess the immunogenicity and safety of a booster dose of MenACWY-CRM vaccine, 10–33 months following the last priming dose in the parent study. The vaccines’ composition has been previously described.^2,3^

The selection of these two studies was facilitated by the availability of remaining aliquots of sera for retest; sera were selected from those obtained by individuals who participated in both studies and had evaluable samples at all timepoints of interest: Visits 3 (pre-second dose for group 2_MenACWY and pre-first dose for groups 1_MenACWY and 1_MenC) and 4 (1 month post-Visit 3) of the parent study and Visits 7 (pre-booster dose) and 8 (1 month post-booster dose) of its extension (Figure 1). The only sera that were *a priori* excluded from this *post-hoc* analysis, irrespective of their availability for retesting, were those obtained from children enrolled in one of the sites from Germany for which noncompliance with Good Clinical Practices was documented during the conduction of the original phase III study. Retest of samples was allowed by the original informed consent obtained from the children’s parents.

Overall, sera from a randomly selected subset of 90 children, who took part in both the parent and the extension study and from whom ≥300 μL of sera were available at each timepoint, were analyzed for antibody responses: sera from 30 children from each of the parent study groups (group 2_MenACWY: priming with 2 MenACWY-CRM doses [at 6–8 and 12 months of age], group 1_MenACWY: priming with 1 MenACWY-CRM dose [at 12 months of age], group 1_MenC: priming with 1 dose of MenC-CRM [at 12 months of age]), who also received a booster dose at 22–45 months of age in the extension study (Figure 1).

### Tested strains

Among the IMD cases observed in Tuscany, 5 hypervirulent MenC strains, isolated by the Laboratory of Immunology and Immunopathology at the Meyer Hospital of Florence during the years 2015 and 2016, were made available for serum bactericidal assay testing.

A genotypic analysis of the ferric enterobactin transport protein A (*f*etA) and porin A (PorA) variable regions was performed.

The phylogenetic network, based on PubMLST^4^ core genome distances, was determined. The distance matrix between isolates was calculated using the BIGSdb Genome Comparator tool,^5^ based on the number of loci carrying different alleles for each pair of strains. The scheme used was *N. meningitidis* cgMLST v1.0, a core genome scheme that includes 1605 loci found to be present in ≥95% *N. meningitidis* isolates.^6^ Neighbor-net diagrams were generated using SplitsTree5.^7^

### Immunogenicity assessment

Antibody titers against the hypervirulent MenC strains were tested using a non-validated serum bactericidal activity (SBA) assay with human complement (hSBA), developed by the Preclinical Evidence Generation Serology Department (GSK, Siena, Italy) and performed, with minor modifications, as previously described.^8^ During the development of the assay, critical parameters for target strains, incubation times, complement sources and test acceptance criteria were defined and reproducibility was demonstrated. The lower limit of quantitation for the hSBA assay was determined as a titer of 1:4.

### Statistical analysis

The analyses were purely descriptive in nature and were not statistically powered to detect differences between groups.

A one-way analysis of variance (ANOVA) model with vaccine group as a factor was used to calculate the hSBA geometric mean titers (GMTs) and geometric mean ratios (GMRs; Visit 4/Visit 3 and Visit 8/Visit 7). The GMTs, GMRs and 2-sided 95% confidence intervals (CIs) were computed by exponentiating (base 10) the least square means and their 95% CIs of the logarithmically transformed (base 10) hSBA titers. Titers below the detection limit (<1:4) were set to half that limit.

GMR was not calculated for group 2_MenACWY as the blood draw at Visit 3 for this group did not correspond to pre-vaccination levels (Visit 3 occurred 4–6 months post-first vaccine dose).

Seroresponse was defined for children with Visit 3 hSBA titer <1:4 as an hSBA titer ≥1:8 at Visit 4, and for children with Visit 3 hSBA titer ≥1:4 as a ≥ 4-fold rise in hSBA titers at Visit 4 (1 month after the first meningococcal vaccine dose for children in group 1_MenACWY and group 1_MenC of the parent study). The associated 2-sided 95% Clopper-Pearson CIs were calculated.

The percentages of children with hSBA titers ≥1:4 and ≥1:8 against the hypervirulent MenC strains and the associated 2-sided 95% Clopper-Pearson CIs were calculated at all timepoints (Visits 3, 4, 7 and 8).

After having randomly selected the 90 children based on sera availability for retesting, a *post-hoc* analysis of the same immunogenicity endpoints against the MenC reference strain used in the 2 clinical studies was performed using the original datasets restricted to the subset of selected children. Bactericidal activity against the reference MenC strain was tested (only in the original trials) with a validated manual hSBA assay^9,10^ at the GSK Clinical Sciences Laboratory (Marburg, Germany).

## Results

### Baseline characteristics for study population

The demographic and other baseline characteristics of the 90 children whose sera were available for retesting are presented in Appendix Table A1 and were similar across groups.

Overall, the mean age (± standard deviation) at time of study enrollment in the parent study was 6.6 ± 0.6 months. Slightly more males than females were included in the groups 2_MenACWY and 1_MenACWY, compared to the group 1_MenC. Across groups, most children (93–100%) were Caucasian.

### MenC strains

**Figure 2.**
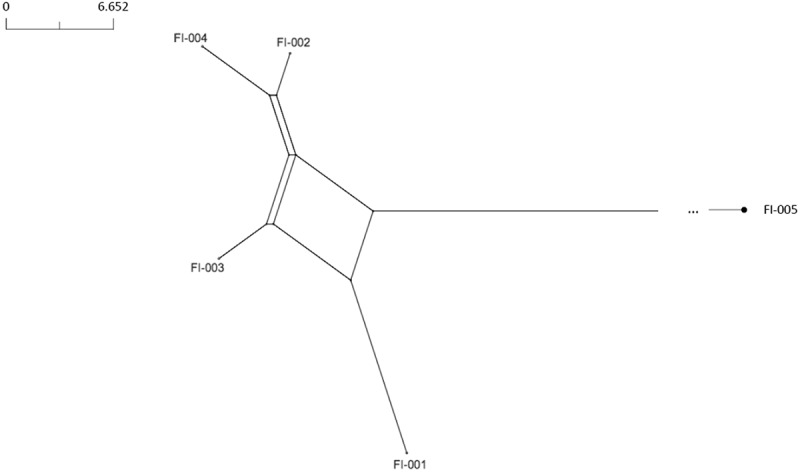
Core genome analysis of the hypervirulent MenC strains

Genetic characterization of the 5 hypervirulent MenC strains showed that 4 of them (FI-001–FI-004) belonged to the C:P1.5–1,10-8:F3-6:ST-11 (cc11) clonal complex, while FI-005 belonged to the C:P1.7–4,14-6:F3-9:ST-1031 (cc334) clonal complex. Four strains (FI-001–FI-004) were almost identical, and distinct from FI-005 (cc334), isolated from sporadic IMD cases (Figure 2). Isolate FI-005 had an average distance of 1430 loci from the other 4 isolates.

### Immunogenicity

#### Geometric mean titers and geometric mean ratios

**Figure 3.**
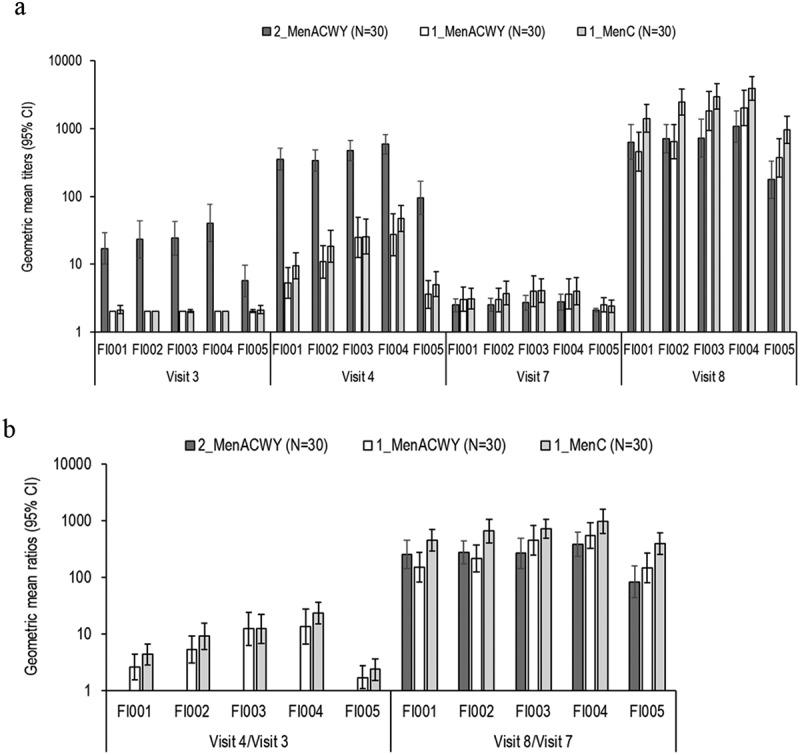
hSBA geometric mean titers (A) and geometric mean ratios (B) for hypervirulent MenC strains

Approximately 1 month after completion of the primary series, hSBA GMTs tended to be lower against the cc334 strain (FI-005, range 3.6–94.8, across groups) than against the other cc11 strains (FI-001–FI-004, range 5.3–588.1, across groups and strains), with the highest titers following 2-dose and the lowest after 1-dose of MenACWY-CRM (Figure 3a). Visit 4/Visit 3 GMRs ranged from 1.7 (FI-005, group 1_MenACWY) to 23.7 (FI-004, group 1_MenC) (Figure 3b).

hSBA, serum bactericidal activity assay with human complement; 95% CI, 95% confidence interval; N, number of children; Group 2_MenACWY, MenACWY-CRM vaccination at 6–8 and 12 months of age; Group 1_MenACWY, MenACWY-CRM vaccination at 12 months of age; Group 1_MenC, MenC-CRM vaccination at 12 months of age.

Before the MenACWY-CRM booster dose (10–33 months post-priming), hSBA GMTs ranged from 2.1 (FI-005, group 2_MenACWY) to 4.1 (FI-003, group 1_MenC) (Figure 3a).

One month post-booster dose, high hSBA GMTs were observed against all five hypervirulent MenC strains in all 3 groups, and especially in the group of children primed with MenC-CRM. hSBA GMTs ranged from 176.9 (group 2_MenACWY) to 955.4 (group 1_MenC) for FI-005 and from 456.1 (FI-001, group 1_MenACWY) to 3911.0 (FI-004, group 1_MenC) for strains belonging to cc11 (Figure 3a). Visit 8/Visit 7 GMRs ranged from 84.4 (group 2_MenACWY) to 397.1 (group 1_MenC) for FI-005, and from 150.5 (FI-001, group 1_MenACWY, FI-001) to 977.8 (FI-004, group 1_MenC) for cc11 strains (Figure 3b).

When tested against the vaccine reference strain (Appendix Table A2), hSBA GMTs were higher in the 2_MenACWY group (279.9), compared to the other 2 (23.9 and 35.6, groups 1_MenACWY and 1_MenC, respectively). Following the MenACWY-CRM booster dose, GMTs were 554.1, 956.0 and 1935.4 in groups 2_MenACWY, 1_MenACWY and 1_MenC, respectively. GMRs ranged from 156.6 (group 1_MenACWY) to 449.2 (group 1_MenC).

#### Seroresponse rates

**Figure 4.**
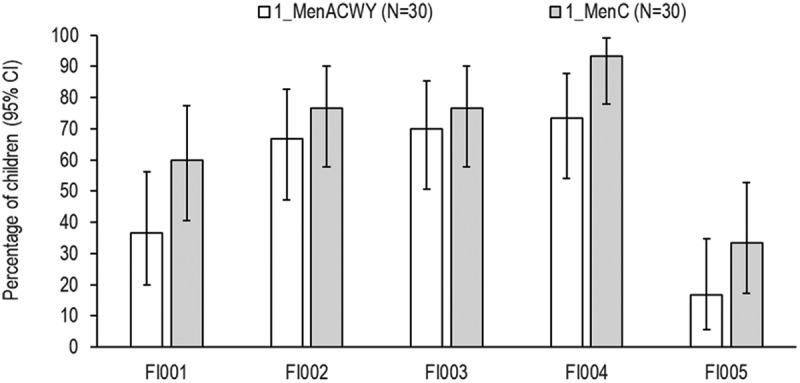
Percentages of children with seroresponse against hypervirulent MenC strains

Seroresponse rates were lower for the FI-005 strain (16.7% and 33.3%, groups 1_MenACWY and 1_MenC, respectively) and higher for the FI004 strain (73.3% and 93.3%, groups 1_MenACWY and 1_MenC, respectively) (Figure 4). When analyzing immune responses against the MenC vaccine reference strain 1 month post-primary series, ≥82.8% of children in groups 1_MenACWY and 1_MenC achieved seroresponse following priming (Appendix Table A2).

95% CI, 95% confidence interval; N, number of children; Group 1_MenACWY, MenACWY-CRM vaccination at 12 months of age; Group 1_MenC, MenC-CRM vaccination at 12 months of age. Seroresponse was defined for children with Visit 3 hSBA titer <1:4 as an hSBA titer ≥1:8 at Visit 4, and for children with Visit 3 hSBA titer ≥1:4 as a ≥ 4-fold rise in hSBA titers at Visit 4.

#### Percentages of children with hSBA titers ≥1:4 and ≥1:8

**Figure A1.**
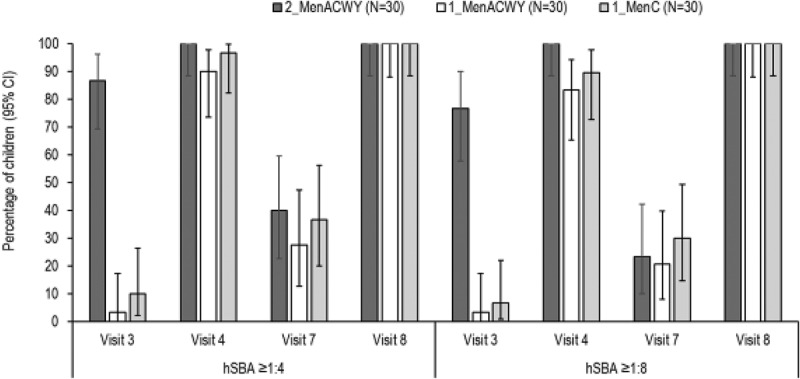
Percentages of children with hSBA titers ≥1:4 or ≥1:8 against the MenC vaccine reference strain (all time points)

One month after completion of the primary series, percentages of children with hSBA titers ≥1:4 ranged from 90.0% (FI005) to 100% (FI001–FI004) in group 2_MenACWY, from 26.7% (FI005) to 80.0% (FI004) in group 1_MenACWY, and from 53.3% (FI005) to 100% (FI004) in group 1_MenC (Table 1). Against the reference MenC strain, similar percentages of children had hSBA titers ≥1:4 across groups, ranging from 90.0% (group 1_MenACWY) to 100% (group 2_MenACWY) (Appendix Figure A1).

Irrespective of the strain tested and the priming schedule used, ≥96.7% of children had hSBA titers ≥1:4 after the MenACWY-CRM booster dose (Table 1). All children showed titers ≥1:4 against the MenC vaccine reference strain.

**Table 1.** Percentages of children with hSBA antibody titers ≥1:4 and ≥1:8 against hypervirulent MenC strains (all time points). hSBA, serum bactericidal activity assay with human complement; %, percentages of children with hSBA titers equal to or above the specified threshold; 95% CI, 95% confidence interval; N, number of children in each group; Group 2_MenACWY, MenACWY-CRM vaccination at 6–8 and 12 months of age; Group 1_MenACWY, MenACWY-CRM vaccination at 12 months of age; Group 1_MenC, MenC-CRM vaccination at 12 months of age; All children were vaccinated with booster dose of MenACWY-CRM at 22–45 months of age. Visit 3 was pre-second dose for group 2_MenACWY and pre-first dose for groups 1_MenACWY and 1_MenC; Visit 4 was post-primary vaccination, Visit 7, pre-booster vaccination, Visit 8, post-booster vaccination

		% (95% CI)					
		2_MenACWY (N=30)		1_MenACWY (N=30)		1_MenC (N=30)	
		hSBA titers ≥1:4	hSBA titers ≥1:8	hSBA titers ≥1:4	hSBA titers ≥1:8	hSBA titers ≥1:4	hSBA titers ≥1:8
FI001	Visit 3	86.7 (69.3; 96.2)	76.7 (57.7; 90.1)	0 (0; 11.6)	0 (0; 11.6)	3.3 (0.1; 17.2)	3.3 (0.1; 17.2)
	Visit 4	100 (88.4; 100)	100 (88.4; 100)	50.0 (31.3; 68.7)	36.7 (19.9; 56.1)	80.0 (61.4; 92.3)	63.3 (43.9; 80.1)
	Visit 7	16.7 (5.6; 34.7)	6.7 (0.8; 22.1)	13.3 (3.8; 30.7)	13.3 (3.8; 30.7)	23.3 (9.9; 42.3)	20.0 (7.7; 38.6)
	Visit 8	96.7 (82.8; 99.9)	96.7 (82.8; 99.9)	96.7 (82.8; 99.9)	96.7 (82.8; 99.9)	100 (88.4; 100)	100 (88.4; 100)
FI002	Visit 3	86.7 (69.3; 96.2)	76.7 (57.7; 90.1)	0 (0; 11.6)	0 (0; 11.6)	0 (0; 11.6)	0 (0; 11.6)
	Visit 4	100 (88.4; 100)	100 (88.4; 100)	70.0 (50.6; 85.3)	66.7 (47.2; 82.7)	86.7 (69.3; 96.3)	76.7 (57.7; 90.1)
	Visit 7	16.7 (5.6; 34.7)	10.0 (2.1; 26.5)	13.3 (3.8; 30.7)	13.3 (3.8; 30.7)	33.3 (17.3; 52.8)	23.3 (9.9; 42.3)
	Visit 8	100 (88.4; 100)	100 (88.4; 100)	100 (88.4; 100)	96.7 (82.8; 99.9)	100 (88.4; 100)	100 (88.4; 100)
FI003	Visit 3	90.0 (73.5; 97.9)	76.7 (57.7; 90.1)	0 (0; 11.6)	0 (0; 11.6)	3.3 (0.1; 17.2)	0 (0; 11.6)
	Visit 4	100 (88.4; 100)	100 (88.4; 100)	76.7 (57.7; 90.1)	70.0 (50.6; 85.3)	86.7 (69.3; 96.2)	76.7 (57.7; 90.1)
	Visit 7	23.3 (9.9; 42.3)	10.0 (2.1; 26.5)	30.0 (14.7; 49.4)	20.0 (7.7; 38.6)	43.3 (25.5; 62.6)	26.7 (12.3; 45.9)
	Visit 8	100 (88.4; 100)	96.7 (82.8; 99.9)	96.7 (82.8; 99.9)	96.7 (82.8; 99.9)	100 (88.4; 100)	100 (88.4; 100)
FI004	Visit 3	93.3 (77.9; 99.2)	90.0 (73.5; 97.9)	0 (0; 11.6)	0 (0; 11.6)	0 (0; 11.6)	0 (0; 11.6)
	Visit 4	100 (88.4; 100)	100 (88.4; 100)	80.0 (61.4; 92.3)	73.3 (54.1; 87.7)	100 (88.4; 100)	93.3 (77.9; 99.2)
	Visit 7	20.0 (7.7; 38.6)	16.7 (5.6; 34.7)	23.3 (9.9; 42.3)	20.0 (7.7; 38.6)	30.0 (14.7; 49.4)	26.7 (12.3; 45.9)
	Visit 8	100 (88.4; 100)	100 (88.4; 100)	100 (88.4; 100)	100 (88.4; 100)	100 (88.4; 100)	100 (88.4; 100)
FI005	Visit 3	40.0 (22.7; 59.4)	40.0 (22.7; 59.4)	3.3 (0.1; 17.2)	0 (0; 11.6)	3.3 (0.1; 17.2)	3.3 (0.1; 17.2)
	Visit 4	90.0 (73.5; 97.9)	90.0 (73.5; 97.9)	26.7 (12.3; 45.9)	20.0 (7.7; 38.6)	53.3 (34.3; 71.66)	36.7 (19.9; 56.1)
	Visit 7	6.7 (0.8; 22.1)	0 (0; 11.6)	13.3 (3.8; 30.7)	10.0 (2.1; 26.5)	13.3 (3.8; 30.7)	6.7 (0.8; 22.1)
	Visit 8	96.7 (82.8 99.9)	96.7 (82.8; 99.9)	96.7 (82.8; 99.9)	96.7 (82.8; 99.9)	100 (88.4; 100)	100 (88.4; 100)

One month post-primary series, the percentages of children with hSBA titers ≥1:8 were in the range of 90.0% (strain FI005) and 100% (all other hypervirulent strains) in group 2_MenACWY, of 20.0% (strain FI005) and 73.3% (strain FI004) in group 1_MenACWY, and of 36.7% (strain FI005) and 93.3% (strain FI004) in group 1_MenC (Table 1). After the primary series, similar percentages of children with hSBA titers ≥1:8 against the reference MenC strain were observed, ranging from 83.3% (group 1_MenACWY) to 100% (group 2_MenACWY) (Appendix Figure A1).

At 10–33 months following the last priming dose, the percentages of children with hSBA titers ≥1:8 were lower than those observed post-vaccination, ranging from 0% to 26.7% before booster administration, and raising to ≥96.7% 1 month post-booster dose against all strains (Table 1). All children had hSBA titers ≥1:8 against the MenC vaccine reference strain after booster administration (Appendix Figure A1).

## Discussion

To our knowledge, this study is among the first to analyze vaccine performance against MenC strains responsible for outbreaks of IMD and the first to assess MenC-CRM/MenACWY-induced bactericidal activity against hypervirulent MenC strains circulating in Italy.

Immune responses against five hypervirulent MenC isolated during an IMD outbreak in Tuscany were evaluated using sera from children previously immunized against MenC. The sera were collected during studies conducted as part of the clinical development program of MenACWY-CRM, which were selected for this *post-hoc* analysis based on several considerations. The first was the interest in exploring the responses in infants and toddlers, who are among the age groups at higher risk for IMD. The second was the potential to evaluate different MenACWY-CRM/MenC-CRM priming schedules and boosting with MenACWY-CRM, in terms of bactericidal activity against hypervirulent MenC strains.

The trigger for this *post-hoc* analysis was the unexpectedly higher incidence of MenC IMD cases observed in 2015–2016 in Tuscany: 0.83 per 100,000 inhabitants in 2015 and 1.98 in the first 2 months of 2016, compared to previous years (an average incidence rate for 2012–2014 of 0.08 per 100,000 inhabitants, and a range from 0.05 in 2014 to 0.11 in 2012).^1,11^ Among the 43 MenC IMD cases reported between January 2015 and February 2016, 5 occurred in individuals who had been vaccinated with a MenC conjugate vaccine. Apart from 1 case in an individual (62 years old) vaccinated on the same day of the symptom onset, in the remaining 4 cases (9–22 years old), the vaccine was administered 2–10 years before the symptom onset. Of the overall 10 cases with fatal outcome, 9 were among those unvaccinated. In 35 out of the 40 samples analyzed, the MenC strain was confirmed as C:P1.5–1,10-8:F3-6:ST-11 (cc11).^1^ A recently published paper reported that the estimated effectiveness of MenC-vaccines was still high (77%; 95% CI: 36–92) during the outbreak, and similar to that calculated for 2006–2016, over the entire period since the introduction of MenC vaccination in Tuscany (80%; 95% CI: 54–92).^11^

In response to this outbreak, an immunization campaign was launched in the region, initially with a single dose of meningococcal tetravalent (MenACWY) conjugate vaccine actively offered to individuals 11–19 years old, even if already vaccinated with MenC conjugate vaccines in childhood. The immunization campaign was then continuously adapted to the evolving epidemiology, for instance extended to older individuals (20–44 years) residing in the area of the local health units with at least 1 reported MenC IMD case, using MenC conjugate vaccines as an alternative option to the tetravalent vaccine (actively offered only to 11–20 years age group).^1^

This *post-hoc* analysis provides a descriptive assessment of antibody responses induced by vaccination with MenC or MenACWY-CRM conjugate vaccines against hypervirulent cc11 or cc334 strains, isolated from MenC IMD cases reported in Tuscany in 2015–2016. Sera were tested with a SBA assay, which is the “gold standard” for measuring the ability of vaccine-induced antibodies to kill *N. meningitidis*.^12,13^ SBA measures complement-mediated killing via antibody and the assay generally uses exogenously added human (hSBA) or rabbit complement (rSBA; an easier source of large volumes of complement without the need for human donors) to mediate killing. For meningococcal vaccines, a generally-accepted correlate of protection is a level of bactericidal antibodies ≥1:8, or even ≥1:4, when using hSBA^14–16^ and ≥1:8 when using rSBA.^17^ In this post-hoc analysis, the hSBA assay was used consistently with the test method used in the original study.

Bactericidal antibody titers varied according to the hypervirulent strain tested and overall resulted in higher titers against the 4 cc11 strains than against the cc334 one, an observation apparently not explained by differences in the capsule composition, as the sialic acid quantitation did not yield significantly different results among the five strains (data not shown). The difference in bactericidal activity may thus rather be related to a different susceptibility to complement-mediated bacterial killing.

When testing sera taken approximately 1 month after priming, immune responses were more robust following 2 doses of MenACWY-CRM, administered 4–6 months apart, than after 1 dose of MenC-CRM or MenACWY-CRM given at 12 months of age, as shown by the percentages of children with putative protective hSBA titers.

Seroresponse rates could not be assessed for group 2_MenACWY, as no blood draw was taken before first MenACWY-CRM dose. For the other two groups, a better performance against the hypervirulent MenC strains was shown when children were primed with MenC-CRM compared to MenACWY-CRM. Similar findings were observed against the MenC vaccine reference strain in the same subset of 90 children, an outcome which could be predicted considering that: i) for group 2_MenACWY, a second dose of the quadrivalent conjugate vaccine was administered 4–6 months apart, maximizing the possibility to mount robust primary responses following a “prime-prime” schedule, compared to those elicited by a single dose of the vaccine, and ii) for group 1_MenC, the MenC antigen content administered per dose was twice as much (10 μg) as MenACWY-CRM (5 μg) and the vaccine contained aluminum hydroxide adjuvant.^2,3^

When analyzing the bactericidal activity after a MenACWY-CRM booster dose, priming with MenC-CRM elicited higher titers compared to either 1- or 2-dose MenACWY-CRM priming. The better performance in terms of anamnestic response following MenC-CRM priming can be explained by the amount of antigen and the presence of the adjuvant in its formulation. Of note, 1 dose of MenACWY-CRM at 12 months tended to result in a better priming than 2 doses of the same vaccine administered at 6–8 and 12 months. However, this should be interpreted with caution, as it may be limited by the selection of the analyzed subset, which was mainly based on availability of sufficient quantities of samples for retesting, in a small group of participants from the clinical trials. Nevertheless, this finding was also observed when restricting the analysis to the MenC vaccine reference strain, an observation that might be biased by the potential effect of the different time interval between the booster dose and the last priming dose, which could vary between 13 and 33 months.

Overall, irrespective of the priming series and the different hypervirulent MenC strain tested, bactericidal titers ≥1:4 and ≥1:8 were detected in sera from almost all children following the booster dose.

This analysis has several limitations, the main being that it was planned and performed *post-hoc*, it is descriptive in nature and based on sera from a limited number of children (N = 90). Another limitation is the design of the original clinical trials, which were the source of the samples for retesting, with a wide chronological range allowed between primary and booster vaccination. In addition, testing against the hypervirulent strains was performed using a non-validated hSBA assay, while the results of the original analysis against the MenC vaccine reference strain were obtained with a validated hSBA assay, thus further preventing any formal comparison.

Notwithstanding these potential limitations, this study brings important information on the performance of meningococcal vaccination against hypervirulent MenC strains. As meningococcal strain circulation and the burden of disease in the field vary across different geographical areas and over time and can hardly be predicted, this analysis may provide insights on possible vaccination strategies in case of hypervirulent MenC outbreaks.

In conclusion, the conjugate vaccines MenACWY-CRM and MenC-CRM were able to elicit immune responses to hypervirulent MenC strains to different extents, depending on the priming series and the strain tested. While 2 MenACWY-CRM doses, administered 4–8 months apart, seemed to provide better priming to infants/toddlers, 1 dose of MenC-CRM at 12 months of age resulted in higher serum bactericidal activity following MenACWY-CRM booster dose administered approximately 1–2 years after. Irrespective of the strain tested and the priming vaccine and schedule adopted, putative protective levels of bacterial antibodies (hSBA titers ≥1:8) were detected in almost all sera collected after the MenACWY-CRM booster dose.

## Figures and Tables

95% CI, 95% confidence interval; N, number of children; Group 2_MenACWY, MenACWY-CRM vaccination at 6–8 and12 months of age; Group 1_MenACWY, MenACWY-CRM vaccination at 12 months of age; Group 1_MenC, MenC-CRM vaccination at 12 months of age.Note: For group 1_MenC, N = 29 for Visit 4.

## Data Availability

The product that is studied in this clinical study, together with the rights to the data and results generated, have been transferred to GSK by Novartis. The results summary for these studies (NCT00667602 and its extension NCT01345721) are available on the Novartis Clinical Trials Results website and can be accessed at https://www.novctrd.com/. For interventional studies that evaluate GSK medicines, anonymized patient-level data are made available to independent researchers, subject to review by an independent panel, at www.clinicalstudydatarequest.com within six months of publication. To protect the privacy of patients and individuals involved in our studies, GSK does not publicly disclose patient level data. Patient-level data for this study will be made available on www.clinicalstudydatarequest.com upon request, subject to any pre-existing rights and obligations and/or consents required under the relevant agreements governing or related to these studies.

## Trademark statement

*Menveo* and *Menjugate* are trademarks owned or licensed by the GSK group of companies.

## Appendix

**Table A1.** Demographic characteristics of patients at the time of enrollment in the parent study

	2_MenACWY (N = 30)	1_MenACWY (N = 30)	1_MenC (N = 30)
Mean age ± SD, months	6.6 ± 0.6	6.5 ± 0.4	6.8 ± 0.8
Female sex, n (%)	13 (43)	13 (43)	16 (53)
Ethnic origin, n (%)			
Caucasian	28 (93)	28 (93)	30 (100)
Asian	1 (3)	1 (3)	0 (0)
Other	1 (3)	1 (3)	
Mean weight ± SD, kg	7.8 ± 1.2	7.9 ± 0.8	8 ± 0.9
Mean height ± SD, cm	68.1 ± 3.0	68.6 ± 2.9	68.2 ± 3.0

N, number of children; SD, standard deviation; n (%), number (percentage) of children in the specified category. Group 2_MenACWY, MenACWY-CRM vaccination at 6–8 and 12 months of age; Group 1_MenACWY, MenACWY-CRM vaccination at 12 months of age; Group 1_MenC, MenC-CRM vaccination at 12 months of age.

**Table A2.** hSBA GMTs, GMRs and seroresponse rates against the MenC vaccine reference strain (all time points)

	Value (95% CI)		
	2_MenACWY (N = 30)	1_MenACWY (N = 30)	1_MenC (N = 30)
Visit 3 GMT	19.7 (12.0; 32.5)	2.1 (1.9; 2.4)	2.6 (1.9; 3.5)
Visit 4 GMT	279.9 (204.0; 384.1)	23.9 (14.7; 39.0)	35.6 (23.5; 53.9)
Visit 4/Visit 3 GMR	-	11.3 (7.0; 18.4)	13.8 (8.2; 23.2)
Visit 7 GMT	3.5 (2.7; 4.7)	3.9 (2.4; 6.2)	4.3 (2.8; 6.6)
Visit 8 GMT	554.1 (352.3; 871.5)	956.0 (580.1; 1575.6)	1935.4 (1321.3; 2834.8)
Visit 8/Visit 7 GMR	156.6 (99.1; 247.6)	247.6 (171.4; 357.6)	449.2 (291.2; 692.8)
Seroresponse rate	-	83.3% (65.3; 94.4)	82.8% (64.2; 94.2)

hSBA, serum bactericidal activity assay with human complement; 95% CI, 95% confidence interval; GMT, geometric mean titer, GMR, geometric mean ratio; N, number of children; Group 2_MenACWY, MenACWY-CRM vaccination at 6–8 and 12 months of age; Group 1_MenACWY, MenACWY-CRM vaccination at 12 months of age; Group 1_MenC, MenC-CRM vaccination at 12 months of age.

Note: Seroresponse was defined for children with Visit 3 hSBA titer <1:4 as an hSBA titer ≥1:8 at Visit 4, and for children with Visit 3 hSBA titer ≥1:4 as a ≥ 4-fold raise in hSBA titers at Visit 4.

For group 1_MenC, N = 29 for the seroresponse rate.
